# Genetic population structures of common scavenging species near hydrothermal vents in the Okinawa Trough

**DOI:** 10.1038/s41598-022-14100-5

**Published:** 2023-02-09

**Authors:** Hiroki Kise, Akira Iguchi, Takuji Ikegami, Yosuke Onishi, Koichi Goto, Yuichiro Tanaka, Travis W. Washburn, Miyuki Nishijima, Taiga Kunishima, Nobuyuki Okamoto, Atsushi Suzuki

**Affiliations:** 1 Geological Survey of Japan, National Institute of Advanced Industrial Science and Technology (AIST), 1-1-1 Higashi, Tsukuba, Ibaraki 305-8567 Japan; 2 Research Laboratory on Environmentally-Conscious Developments and Technologies [E-Code], National Institute of Advanced Industrial Science and Technology (AIST), Tsukuba, 305-8567 Japan; 3 KANSO TECHNOS CO., LTD., Chuo-ku, Osaka, 541-0052 Japan; 4 Wakayama Prefectural Museum of Natural History, 370-1 Funoo, Kainan, Wakayama 642-0001 Japan; 5 Japan Oil, Gas and Metals National Corporation (JOGMEC), Minato-ku, Tokyo, 105-0001 Japan

**Keywords:** Ecological genetics, Ecological genetics

## Abstract

Deep-sea mining of hydrothermal deposits off the coast of Japan is currently under consideration, and environmental baseline studies of the area are required to understand possible impacts. The aim of this study is to clarify population structures of dominant benthic megafaunal species near hydrothermal vent fields in the Okinawa Trough, using a population genetics approach. We examined dominant deep-sea scavenging species including eels, several amphipods, and a decapod and performed population genetic analyses based on the mitochondrial cytochrome *c* oxidase subunit I region. Several sites were sampled within Okinawa Trough to examine intra-population diversity while two other locations 1400–2400 km away were chosen for inter-population comparisons. For synaphobranchid eels *Simenchelys parasitica* and *Synaphobranchus kaupii*, our results showed significant intra-population diversity but no inter-population genetic differentiation, suggesting strong genetic connectivity and/or large population sizes. In addition, single nucleotide polymorphism analysis also confirmed strong genetic connectivity for *Simenchelys parasitica*. Among scavenging amphipods, we detected seven putative species using molecular phylogenetic analysis. We evaluated population structures of the most abundant species of amphipods and a decapod species (*Nematocarcinus lanceopes*). Our results provide basic information on the genetic population structures of benthic megafaunal species near hydrothermal vent fields, which can be used to select candidate species for future connectivity analysis with high-resolution genetic markers and aid understanding of the potential population impacts of environmental disturbances.

## Introduction

Resource developers and those involved in biodiversity conservation have raised numerous concerns about the extraction of seafloor minerals^1^. One such resource, seafloor massive sulfides (SMS), is associated with deep-sea hydrothermal vents^2–4^. However, the chemosynthetic environments of hydrothermal ecosystems have fostered endemic fauna that are also a potential target for conservation. While vent communities themselves have been studied extensively, the marine fauna near hydrothermal vent fields have received little attention in comparison^5^. The exploitation of SMS is likely to release suspended particles and heavy metals that will not only affect vent communities but also fauna near hydrothermal vent fields that are not directly associated with them, hereafter referred to as “near-vent organisms”. The potential effects of such a scenario are still not well understood.

To achieve a stable supply of mineral resources for the country, the Japan Oil, Gas and Metals National Corporation (JOGMEC) is exploring the extraction of SMS resources, including environmental impact assessments from mining activities. Previous studies have suggested that deep-sea mining of SMS may result in decreases in biodiversity through removal of habitat, release of toxic metals, and burial of organisms from sedimentation [e.g.,^5–7^], among other impacts. To understand environmental impacts from exploitation of SMS, JOGMEC started a technical survey project in 2008 to explore massive sulfide deposits in a seafloor depression near Okinawa in southwest Japan^8^. JOGMEC performed multiple biological, physical, and geochemical surveys in accordance with guidance provided by the International Seabed Authority^9^. This included a baseline survey to collect near-vent organisms from the Okinawa Trough.

Information on genetic diversity and connectivity patterns (i.e., determining genetic population structure) is useful for estimating how populations of near-vent organism communities could recover from the impact of mineral extraction^10^. Megabenthos are a key component of benthic communities, and there have been a relatively large number of studies using population genetic analyses of megabenthos inhabiting hydrothermal vents [e.g.,^11^]. Because hydrothermal vent distributions are variable and distances between vents can be far apart, it has been suggested that megabenthos inhabiting hydrothermal vents have considerable dispersal ability^12–14^. On the other hand, studies on population genetics of near-vent organisms are still limited. Therefore, understanding the population structure of near-vent organisms, in combination with studies on vent organisms provides important insights into the formation and maintenance mechanisms of hydrothermal ecosystems and their surrounding environments as well as for assessing the impacts of future resource exploitation.

Given the above context, we performed population genetic analyses of several dominant scavenging near-vent megabenthos in the Okinawa Trough based on the mitochondrial cytochrome *c* oxidase subunit I (COI) region. The mitochondrial COI region is used for relatively high-resolution analysis of interspecies- and intraspecies-level structure^15^, and it is often used for comparing genetic population structures among deep-sea species^11,16^. Such comparisons are informative to infer whether a species is sensitive to environmental disturbances as small population/species ranges are generally associated with higher sensitivity. We also conducted population analyses using larger sets of single nucleotide polymorphisms (SNPs) for a single eel species to provide greater resolution and a more comprehensive understanding of genetic structure. Finally, in conjunction with our findings we discuss possible future research efforts targeting near-vent organisms needed for SMS mining to occur.

## Materials and methods

### Sampling and DNA extraction

**Figure 1:**
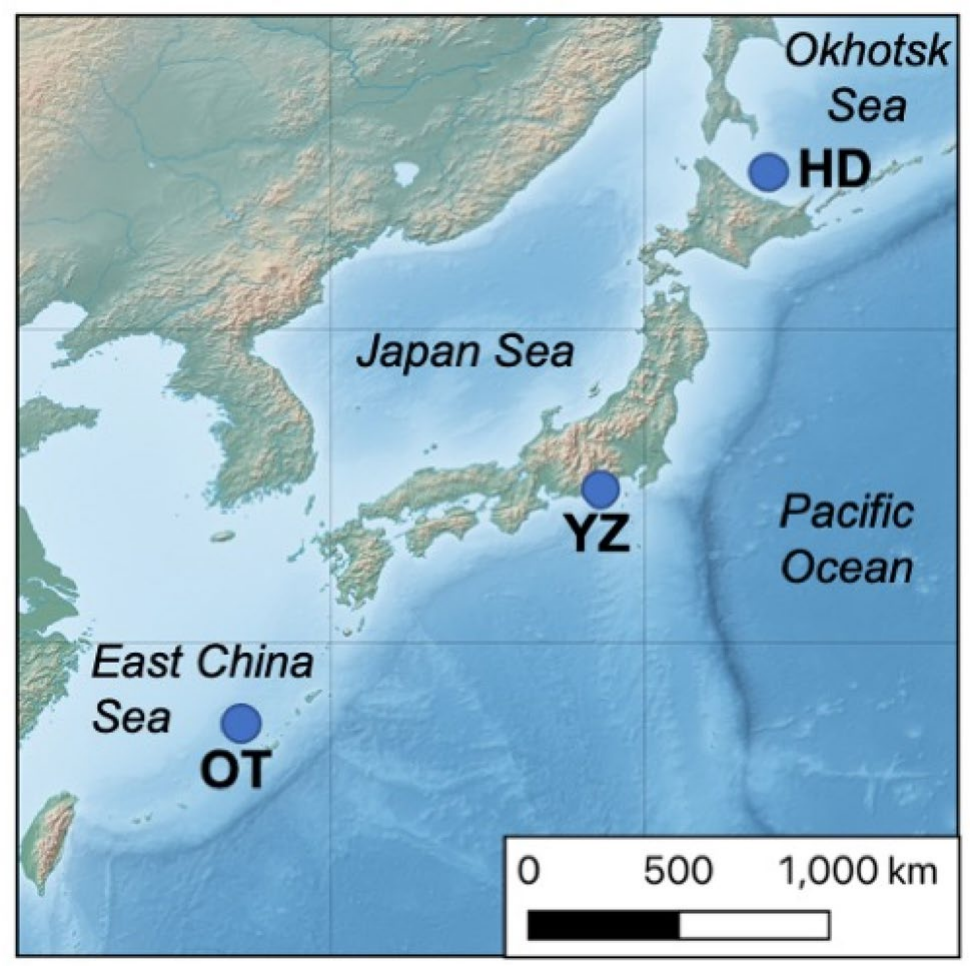
Map showing sampling sites off of Japan. OT = Okinawa Trough, YZ = Suruga Bay, and HD = offshore Hokkaido. This map was made with Natural Earth (free vector and raster map data, https://www.naturalearthdata.com, 1:10 m Cross-blended Hypsometric Tints, version 2.0.0) using the free and open source QGIS version 3.18.1 (https://qgis.org/en/site/).

We collected specimens of several deep-sea benthic scavengers including two synaphobranchid eel species, *Simenchelys parasitica* Gill, 1879^17^ (n = 102) and *Synaphobranchus kaupii* Johnson, 1862^18^ (n = 6), one decapod species, *Nematocarcinus lanceopes* Spence Bate, 1888^19^ (n = 23), and multiple amphipod species (n = 43) using baited traps (shrimp pot, conger tube) with Pacific saury during cruises conducted to carry out a technical survey of seven locations in the Okinawa Trough (OT) between 2013 and 2015 (Fig. 1 and Table 1). Decapods were attracted by baited traps and collected with a sledge net. *Si. Parasitica*, *Sy. kaupii*, and *N. lanceopes* were first identified based on morphology. Collected specimens were preserved in a – 20 ºC freezer on board and upon return to land before experiments.

**Table 1:** Summary of specimens used in this study, the areas where they were collected, and Accession numbers associated to their DNA entries in DNA Data Bank of Japan database. The average distance between the different samples collected in Okinawa (OT) is 3.29 km, and the distance range is 0.02–7.90 km. The average depth for the whole set of samples analyzed in this study (YZ + HD + OT) is 1520 m, and the depth range is 840–1628 m.

Taxonomy	Collected year	Region	Site	Number of individuals	Accession no
*Simenchelys parasitica*	2014	Okinawa Trough	OT1	20	LC532948-LC532967
	2014	Okinawa Trough	OT2	20	LC532968-LC532987
	2014	Okinawa Trough	OT3	20	LC532988-LC533007
	2014	Okinawa Trough	OT4	20	LC533014-LC533033
	2014	Okinawa Trough	OT5	14	LC533034-LC533047
	2015	Okinawa Trough	OT6	2	LC533048-LC533049
	2015	Okinawa Trough	OT7	6	LC533008-LC533013
	2015	Suruga Bay	YZ	24	LC533050-LC533073
*Synaphobranchus kaupii*	2014	Okinawa Trough	OT4	2	LC532911-LC532912
	2014	Okinawa Trough	OT1	1	LC532913
	2015	Okinawa Trough	OT6	3	LC532914-LC532916
	2015	Suruga Bay	YZ	6	LC532917-LC532922
	2016	Off Hokkaido	HD	25	LC532923-LC532947
Amphipoda spp.	2014	Okinawa Trough	OT2	29	LC532844-LC532872
	2013	Okinawa Trough	OT5	11	LC532874-LC532876, LC532879-LC532887
	2014	Okinawa Trough	OT5	3	LC532873, LC532877-LC532878
*Nematocarcinus lanceopes*	2015	Okinawa Trough	OT7	23	LC532888-LC532910

For the two synaphobranchid eel species, we also examined specimens from two regions outside the Okinawa Trough using organisms from markets to estimate connectivity across large geographic distances; offshore of Hokkaido (HD; n = 25 for *Sy. kaupii*) and Suruga Bay (YK; n = 6 for *Sy. kaupii*, n = 24 for *Si. parasitica*) near Shizuoka Prefecture, Japan (Fig. 1 and Table 1). Specimens of *Sy. kaupii* from Hokkaido were purchased in a fish market (collected nearby in the Sea of Okhotsk). The distances are ~ 1400 km between the Okinawa Trough and Suruga Bay and ~ 2400 km between the Okinawa Trough and Hokkaido.

DNA extraction was performed using the DNeasy Blood and Tissue kit (QIAGEN, Hilden, Germany) from tissues of preserved specimens according to the manufacturer’s protocol. Extracted DNA was checked by NanoDrop (ThermoFisher Scientific, Waltham, MA, USA) and quantified by Qubit dsDNA HS assay kit (ThermoFisher Scientific, Waltham, MA, USA).

### PCR and sequencing

To determine mitochondrial COI sequences, we performed PCR by 20 μL mixture containing 0.5 or 1.0 μL of DNA template, 0.5 μL forward primer (20 μM), 0.5 μL reverse primer (20 μM), 1.6 μL dNTP, 2 μL 10X ExTaq buffer, 0.1 μL ExTaq HS (Takara Bio Inc., Otsu, Japan), and 14.8 or 14.3 μL distilled water. We first used universal primers LCO1490 and HCO2198 and PCR condition: 30 cycles of 0.5 min at 94 °C (denaturation), 1 min at 50 °C (annealing), and 1.5 min at 72 °C (extension), followed by an additional extension for 5 min^20^. PCR extensions failed for eels, so we designed new primer sets (Table S1) based on a mitogenome sequence of *Si. parasitica* (accession no. NC_013605). The PCR conditions are as follows: 1 min at 94 °C, 35 cycles of 0.5 min at 94 °C (denaturation), 0.5 min at 60 °C (annealing), and 1 min at 72 °C (extension), followed by an additional extension for 10 min. For amphipods, in addition to universal primers above, we used GrajapCOIF and GrajapCOIR^21^ and a new primer set (Table S1) designed manually on the basis of a mitochondrial COI sequence of Lysianasoidea sp. (accession no. EF989712). PCR conditions are as follows: 1 min at 94 °C, 35 cycles of 0.5 min at 94 °C (denaturation), 0.5 min at 45 °C (annealing), and 1 min at 72 °C (extension), followed by an additional extension for 10 min.

Each PCR product was cleaned with ExoSAP-IT (Affymetrix, Santa Clara, CA, USA) following the manufacturer’s protocol for direct sequencing. The primers used for the sequencing were the same as those for the PCR amplification (both forward and reverse primers). The purified PCR products were sequenced using the ABI 3730xl DNA Analyzer (Applied Biosystems, CA, USA). Subsequently, partial sequences of COI were obtained through checking the DNA chromatograms by eye and used for the analyses below. Sequence identity was confirmed by NCBI BLASTN. Nucleotide sequences were translated into amino acid sequences to check for the presence of stop codons. Sequence data obtained in this study were deposited in the DNA Data Bank of Japan with the accession nos. LC532844–LC533073.

For *Simenchelys parasitica*, SNPs were obtained using the protocol of multiplexed ISSR genotyping by sequencing (MIG-seq)^22^ from 12 to 16 specimens from each site (Table S2). MIG-seq is known as a relatively easy method to evaluate SNPs^22^. Briefly, we amplified regions of genome DNA around inter-simple sequence repeat (ISSRs) by using universal primer pairs (MIG-seq primer set 1) for the 1st PCR. Then, we pooled DNA libraries with different indexes added by the 2nd PCR and sequenced by DNBSEQ-G400 (MGI Tech.) as paired-end reads (2 × 100 bp). All fastq files have been deposited in the DDBJ database (accession no. DRA014289).

### Bioinformatics

For all species examined in this study, we processed sequence data by using custom R code, software version 4.0.3^23^, running packages including *ape* [functions: read.dna, dist.dna, haploNet;^24^ and others maintained by the Bioconductor project (https://www.bioconductor.org/), after alignment with MAFFT v7.402^25^ with the default settings in each species. We also used SeqKit Version: 0.8.1^26^ to obtain basic information (sequence length, the number of sequences) on the FASTA files used in this study. We constructed haplotype networks and calculated genetic diversities by using the R package *pegas* [functions: haplotype, hap.div, nuc.div;^27^ under default settings. We also performed analysis of molecular variance (AMOVA) with 1,000 permutations based on R package *pegas* [function: amova;^27^ using two (Okinawa Trough and Suruga Bay) populations of *Si. parasitica* (specimens of OT1–OT7 collected in 2014 and 2015 were pooled as one population because these were all within ~ 5 km of one another; Table 1) and three populations (Okinawa Trough, Suruga Bay, and offshore of Hokkaido) of *Sy. kaupii* (again all samples in the Okinawa Trough were pooled as a single population; Table 1).

For deep-sea amphipods, Neighbor-Joining (NJ) trees were constructed on genetic distances of Kimura 2-parameter models by using MEGA 7^28^ with 1,000 bootstrap replicates. A model test was performed using ModelTest-NG version 0.1.6^29^, and maximum likelihood (ML) analysis was performed using RAxML-NG version 1.0.3^30^ with 1,000 bootstrap replicates under a GTR (General Time Reversible) + G + I model. Bayesian inference (BI) of phylogenetic analysis was performed using MrBayes v3.2.7^31^ under a GTR + G + I model and 1 MCMC chain (1,000,000 generations and 300,000 for burn-in at which the average standard deviation of split frequency was steadily below 0.01). To identify molecular operational taxonomic units (MOTUs) of deep-sea amphipods, we used Assemble Species by Automatic Partitioning (ASAP)^32^ based on K80 distance model.

For *Si. parasitica*, we filtered reads obtained by MIG-seq with FASTX-Toolkit (http://hannonlab.cshl.edu/fastx_toolkit/index.html) using a fastq-quality-filter v0.0.13 (–Q 33 –q 30 –p 40). Adapter sequences were removed by Cutadapt v2.5^33^. Then we removed shorter reads (< 40 bp) using Seqkit v0.9.1^26^. SNPs were called using the denovo_map.pl pipeline implemented in Stacks v2.0^34^. The parameters used in stacks were as follows: ustacks (-m 3 -M 4) and cstacks (-n 4). Then, we made genepop files using populations (–min-maf 0.03 –max-obs-het 0.5, and -r 0.70) of Stacks. Biallelic loci were filtered with Plink version 1.9^35^ and loci showing deviation from Hardy–Weinberg equilibrium (*p* < 0.001) and minor allele frequencies (–maf 0.03) were filtered. We obtained basic parameters of genepop files using the package “adegenet” v2.1.3^36^ in R v4.0.3^23^. Principal component analysis (PCA) was also performed with a matrix of individual genotype frequencies using R package “hierfstat”^37^. In addition, Permutational Multivariate Analysis of Variance (PERMANOVA) with 1st–4th PCs based on Euclidean distances and 999 permutations was performed using the adonis function in the R package vegan^38^.

## Results

We obtained a 767-bp COI sequence of *Simenchelys parasitica* (BLASTN top hit to nt database: the same species, accession no. AP010849, identity: 99%); a 591-bp sequence from *Synaphobranchus kaupii* (BLASTN top hit to nt database: the same species, accession no. JF952873, identity: 99%); and a 669-bp sequence from *Nematocarcinus lanceopes* (BLASTP top hit to nr database: the same species, accession no. ABQ43464, identity: 99%).

**Figure 2:**
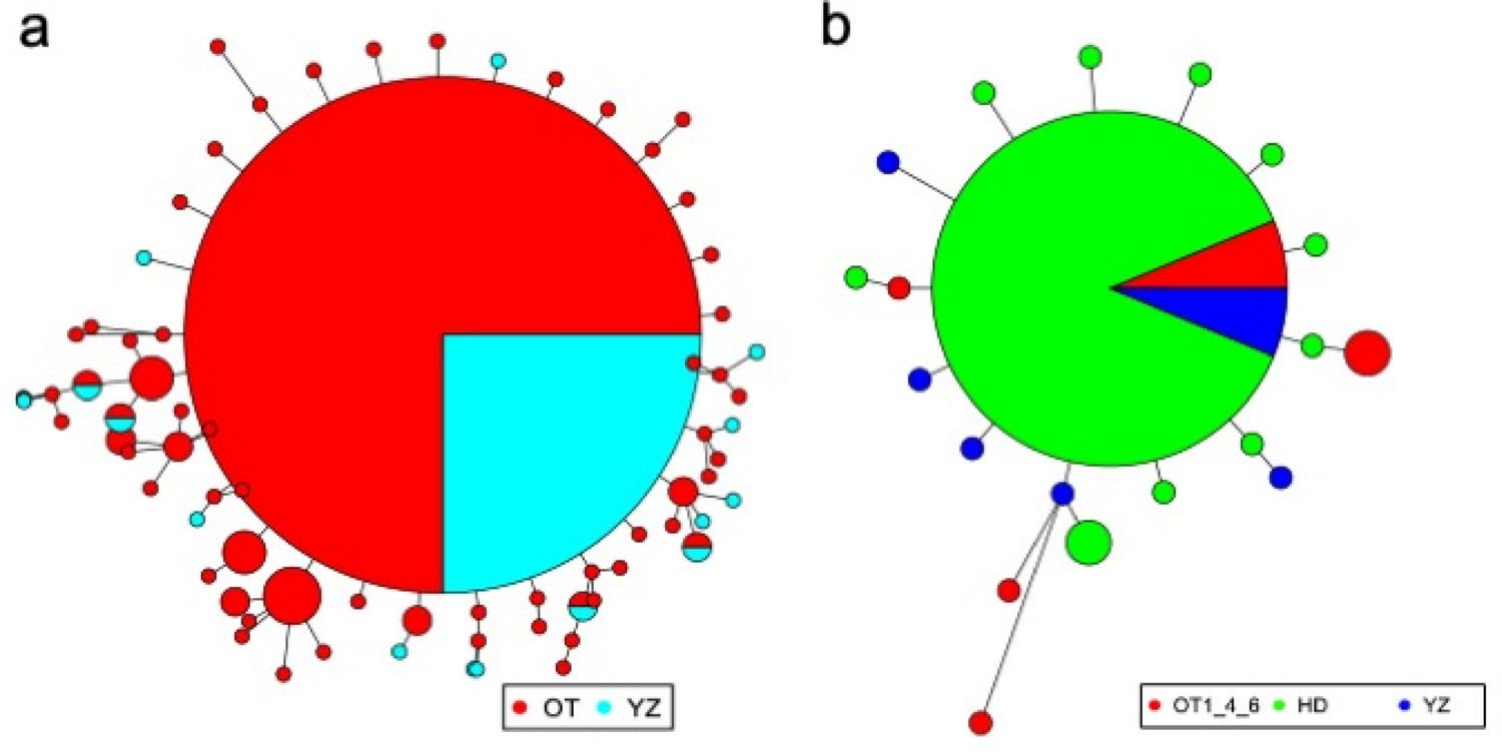
Haplotype networks of synaphobranchid eels. (**a**) *Simenchelys parasitica*. (**b**) *Synaphobranchus kaupii*. Each color corresponds to a population source.

**Figure 3:**
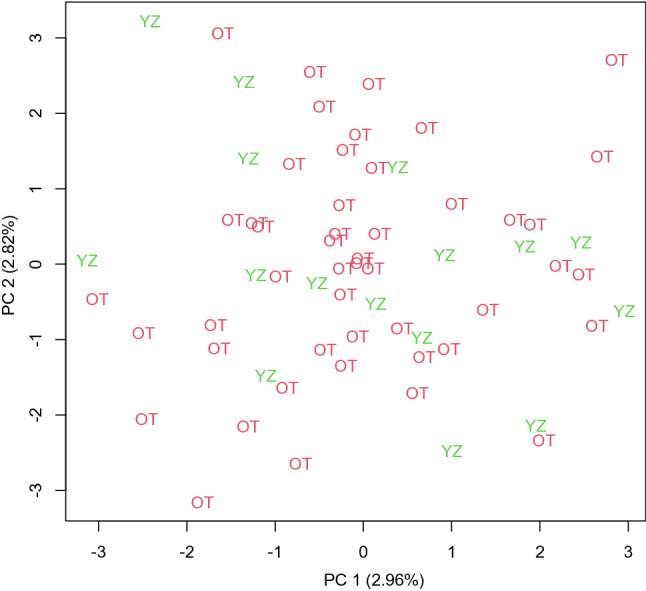
Plot of 1st and 2nd axes of principal component analysis based on a matrix of individual genotype frequencies of *Simenchelys parasitica*. Abbreviations; Okinawa Trough (OT), Suruga Bay (YZ).

Our data indicated high genetic diversity in *Si. parasitica* populations (Okinawa Trough and Suruga Bay; haplotype diversity = 0.92; Table 2) as well as within population (haplotype diversity > 0.85; Table 3). The AMOVA results indicated that there was no significant genetic differentiation among populations of *Si. parasitica* (*p* = 0.4675; Table S3). We also obtained genetic information on three populations of *Sy. kaupii* (Okinawa Trough, Suruga Bay and Hokkaido; Tables 2, 4) and found relatively low genetic diversity in Hokkaido (haplotype diversity = 0.69; Table 4), while high genetic diversity was found within the other two sites (haplotype diversity > 0.93; Table 4). The AMOVA results showed no significant genetic differentiation among *Sy. kaupii* populations (*p* = 0.4805; Table S3). Haplotype networks showed a star-like topology indicating rapid range expansion in both synaphobranchid eel species (Fig. 2). In addition, the MIG-seq analysis detected 110 SNP loci that are available for population genetic analyses for *Si. parasitica* after filtering random biallelic loci with Plink. PCA indicated no grouping among different populations (Fig. 3) based on individual genotype frequencies. Furthermore, PERMANOVA did not find any significant differences of PCAs among five sites (pseudo *F* = 1.299, *p* = 0.201).

**Table 2:** Summary of genetic analysis results of non-vent animals across populations.

Taxonomy	Number of individuals	Number of populations	Number of haplotypes	Haplotype diversity	Nucleotide diversity
*Simenchelys parasitica*	126	7	75	0.9173333	0.003610852
*Synaphobranchus kaupii*	37	3	19	0.8168168	0.004026986
Amphipoda spp.	43	–	19	–	–
Amphipoda sp. (Clade I of Fig. 5)	25	1	7	0.43	0.001116928
*Nematocarcinus lanceopes*	23	1	11	0.798419	0.002235418

**Table 3:** Summary of genetic analysis results of *Simenchelys parasitica* within populations.

Site	Number of individuals	Haplotype diversity	Nucleotide diversity
OT1	20	0.9684211	0.003918205
OT2	20	0.8526316	0.003684897
OT3	20	0.9842105	0.003801551
OT4	20	0.9210526	0.003019282
OT5, OT6	16	0.95	0.003780965
OT7	6	0.9333333	0.002172968
YZ	24	0.8695652	0.003906619

**Table 4:** Summary of genetic analysis results of *Synaphobranchus kaupii within populations.*

Site	Number of individuals	Haplotype diversity	Nucleotide diversity
OT1, OT4, OT6	6	0.9333333	0.0106035
HD	25	0.6933333	0.002278912
YZ	6	1	0.004512126

**Figure 4:**
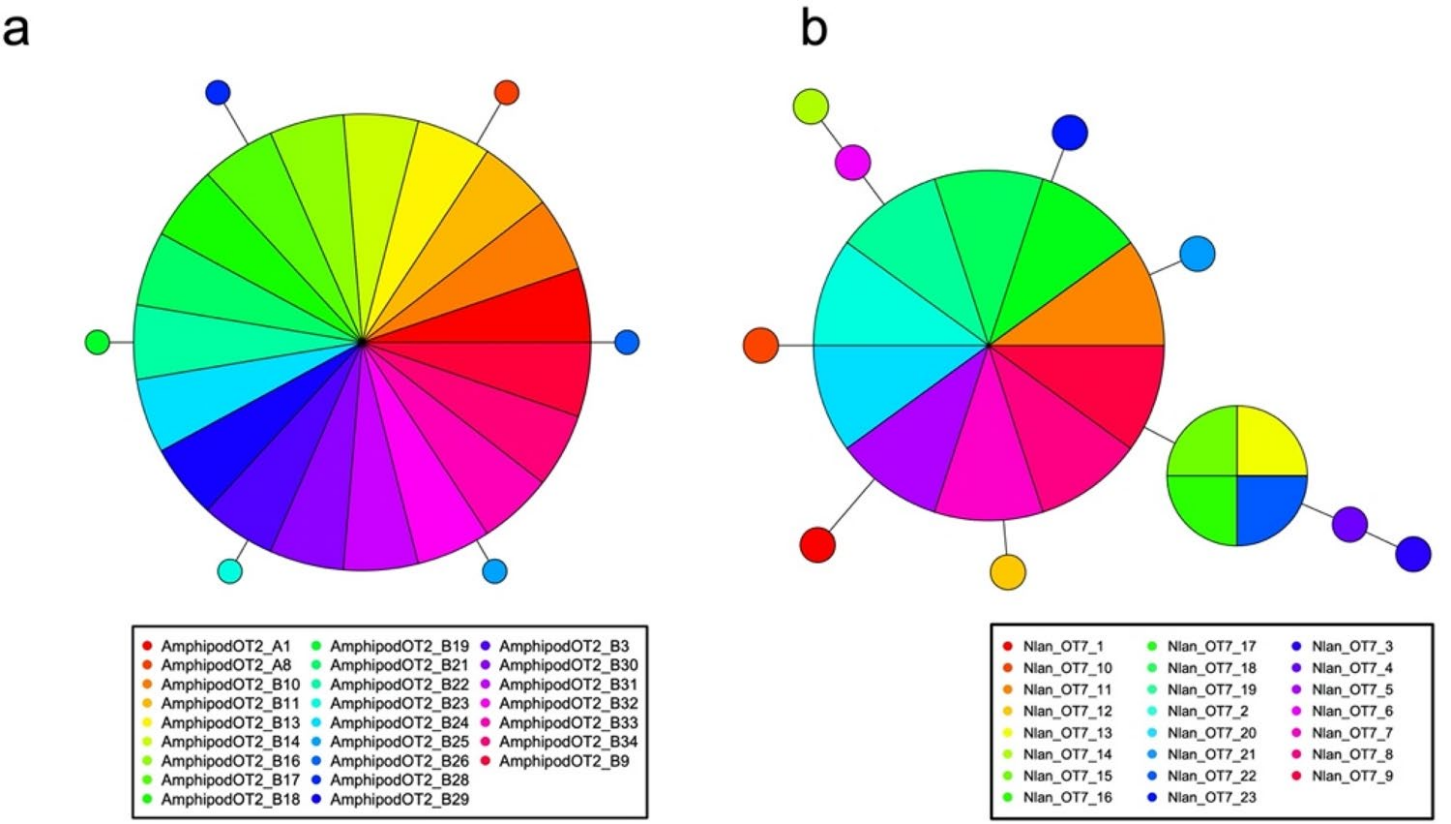
Haplotype networks of sampled benthos, including (**a**) Amphipoda sp. (Clade I of Fig. 5), (**b**) *Nematocarcinus lanceopes*. Each color corresponds to an individual source.

The 23 sequences of *N. lanceopes* included 11 haplotypes (Table 2; BLASTP top hit to nr database: *N. lanceopes*, accession no. ABQ43464, identity: 99%). The level of genetic diversity was relatively moderate (haplotype diversity = 0.80, Table 2), and the haplotype network also showed a star-like topology (Fig. 4b).

**Figure 5:**
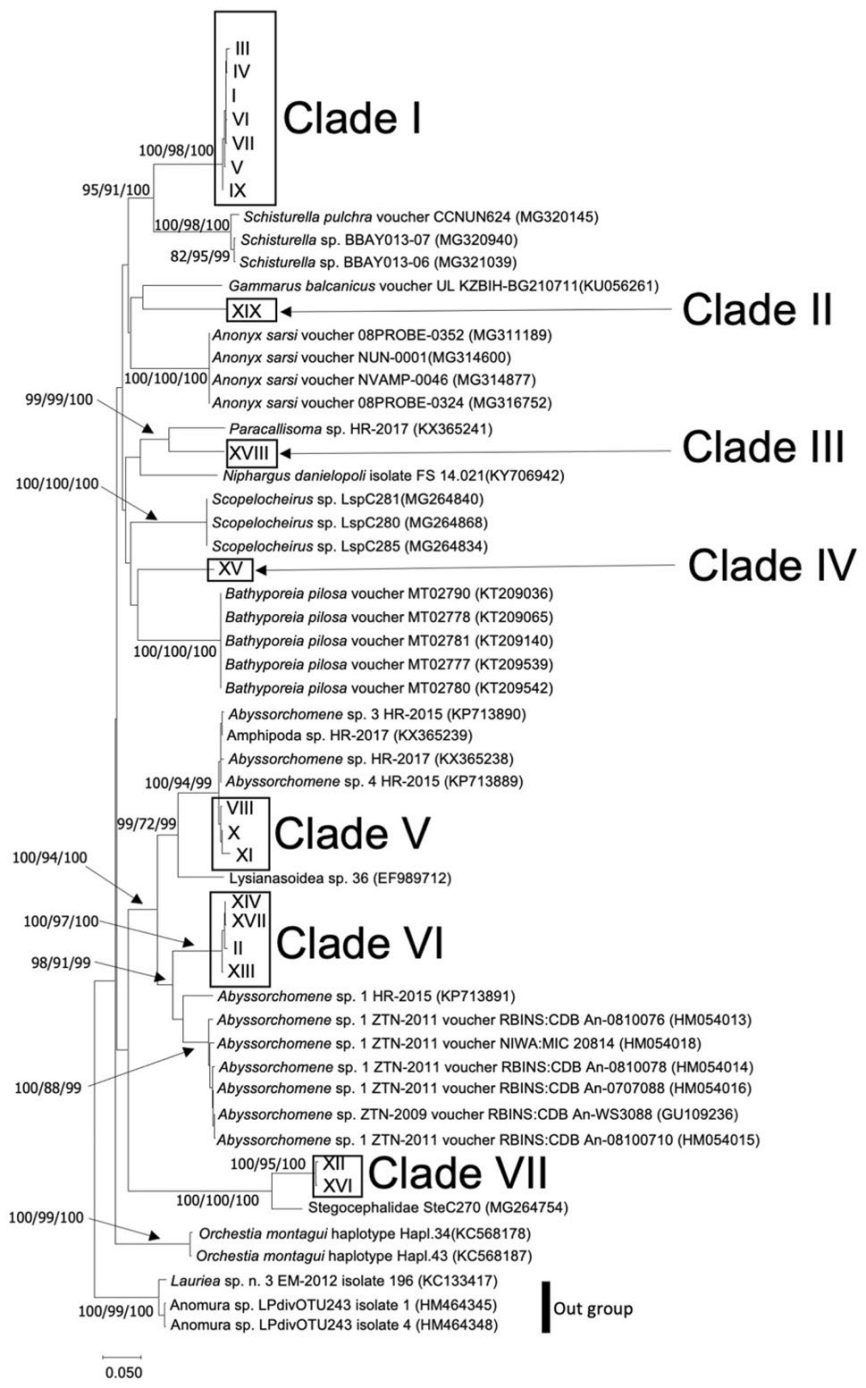
Molecular phylogenetic tree of amphipod species. Numbers indicate bootstrap values of neighbor-joining and maximum likelihood methods (only those > 70% are shown) and Bayesian posterior probabilities (shown as NJ/ML/BI). Boxes show clades containing the haplotypes we obtained. Scale indicates 0.05 substitutions per site.

The amphipod specimens seemed to include several species. Therefore, after extracting 19 haplotypes from 43 sequences (570–573-bp; Table 2, S4), we performed BLASTN with all haplotypes to nt database. The results indicated that 13 haplotypes belonged to the superfamily Lysianassoidea Dana, 1849^39^ (Table S5). We used the similar sequences of each haplotype to perform a molecular phylogenetic analysis, the results of which suggested the existence of seven putative species, as supported by high bootstrap probabilities (NJ =  > 99%, ML =  > 94%, BI =  > 99%; except for Clades II and IV; Fig. 5) and ASAP (asap-score = 1.50). We grouped 25 sequences that putatively belonged to a single species resembling *Schisturella pulchra* (Hansen, 1888)^40^ (Clade I, Fig. 5; Table S5) into one population in the Okinawa Trough, showing relatively low genetic diversity (haplotype diversity = 0.43; Table 2). We also constructed a haplotype network for Clade I; the network had a typical star-like topology (Fig. 4a).

## Discussion

Much attention has been given to active deep-sea hydrothermal vents and the unique communities present in these habitats^13,41,42^, but it is important to evaluate connectivity not only for vent organisms but also for the common deep-sea biota that inhabit the hydrothermal vicinity or “near-vent” organisms. In this study, we described the genetic population structures of several dominant megabenthic organisms (two synaphobranchid eels, one decapod shrimp, and multiple scavenging amphipods) near-vent fields attracted by baited traps. While the species attracted by baited traps may make up a small percentage of the diversity in an area, they can represent a large majority of the abundance as measured with trawl catches^43^. Synaphobranchid eels are dominant components of the scavenging community in the deep sea^44–46^. We performed genetic analyses on populations of far-flung synaphobranchid eel populations > 2000 km apart, but we could not detect significant differentiation in either eel species among the various sites. This indicates that both species may disperse over large distances (over 1000 km through several generations) and maintain large populations. Some *Synaphobranchus* species possess polycyclic ovaries^47^, suggesting frequent opportunities for sexual reproduction among their congeners, which would increase the opportunity for random mating by many individuals. In addition, it is reported that *Synaphobranchus kaupii* shows high swimming and metabolic activity^48^, as this species has even been found in abundance in different ocean basins^49^. Thus, these strategies on life history may serve to increase genetic exchange among populations.

Deep-sea shrimps are also often attracted by baited cameras, and we collected and analyzed 23 individuals of the species *Nematocarcinus lanceopes.* The haplotype network of *N. lanceopes* showed a typical star-like topology (Fig. 4b), but the topology was more complicated than those of the other species networks we examined. Such interspecific differences of population structure are also reported from hydrothermal vent shrimp species in the same area^11^. Future studies should evaluate more individuals and populations to infer whether these topological patterns apply to this species. Population genetic analysis using microsatellite markers has also been attempted in *N. lanceopes*^50–52^, but the target populations in previous studies were obtained around Antarctica. It is necessary to verify beforehand whether the target population is the same as our target species and whether the same microsatellite markers can be applied.

Roughly 80% of the amphipods examined in this study belonged to the superfamily Lysianassoidea, which is ubiquitous in the deep sea^53^. Haplotypes VIII, X, and XI in Clade V (Fig. 5) were similar to Amphipoda sp. (accession no. KX365239), but the neighboring haplotypes (accession nos. KX365238, KP713889, and KP713890) were all *Abyssorchomene*. Thus, these haplotypes likely belong to the genus *Abyssorchomene*. Our samples contained relatively large numbers of individuals in Clades I and VI (25 and 7 individuals, respectively). Therefore, these two amphipod clades can be considered as good targets for future connectivity analysis focused on organisms near hydrothermal vent fields, at least those in Okinawa Trough. The availability of a relatively large number of amphipod samples makes them suitable for conducting reliable population genetic analyses. Our population analysis of individuals in Clade I of amphipod (Fig. 5), which was similar to *Schisturella pulchra* (Table S5), showed relatively low genetic diversity among our samples (Table 2). It should be noted that this is likely partly the result of examining only one population. Thus, further studies including additional sites are needed to evaluate the geographic patterns in genetic diversity in this amphipod clade. It should also be noted that the degree of genetic differentiations does not always equate to dispersal abilities among species and is often affected by population history (e.g.^16^). Future studies of movement patterns, either by swimming or by passive larval dispersal, would be useful to explain the population structures we identified in this study.

In this study, we mainly used COI sequences to clarify basic information on the genetic population structures of organisms near hydrothermal vent fields. It is important, however, to be cautious when interpreting our results. While there have been numerous studies that have based their results and conclusions on COI sequences including for deep-sea species^11,16^, the genetic marker we chose was not as variable as those commonly used for coastal marine organisms^54^. In future studies, analyses using highly variable genetic markers such as microsatellites^55^ and single nucleotide polymorphisms (e.g. MIG-seq used in this study) should be used to detect more detailed population structures. Highly polymorphic markers have already been used in deep-sea amphipods^55,56^, and it is hoped that similar analyses will be applied to near-vent amphipods from this study in the future. Such information on genetic connectivity would be essential in assessing the environmental impacts of mineral mining around hydrothermal vents, as well as to determine potential locations of deep-sea marine protected areas^57,58^. Although a trial has been conducted to infer community resilience in hydrothermal vents considering a dispersal network^10^, the connectivity data supporting this dispersal network is still limited, thus it is necessary to accumulate connectivity data for hydrothermal vents and their surrounding ecosystems in the future.

## Supplementary Information

Supplementary Information.

## Data Availability

All sequences and fastq files were deposited in DNA Data Bank of Japan database (LC532844-LC533073, DRA014289).

## References

[1] Van Dover, C. L. *et al. *Environmental management of deep-sea chemosynthetic ecosystems: justification of and considerations for a spatially based approach. ISA Technical Study: No.9. (International Seabed Authority, 2011).

[2] Ikehata, K., Suzuki, R., Shimada, K., Ishibashi, J., & Urabe, T. Mineralogical and Geochemical Characteristics of Hydrothermal Minerals Collected from Hydrothermal Vent Fields in the Southern Mariana Spreading Center. In S*ubseafloor biosphere linked to hydrothermal systems: TAIGA Concept*. 275–288 (Springer Tokyo, 2015).

[3] Rona PA, Scott SD (1993). A special issue on sea-floor hydrothermal mineralization; new perspectives; preface. Econ. Geol..

[4] Glasby GP, Iizasa K, Yuasa M, Usui A (2000). Submarine hydrothermal mineralization on the Izu-Bonin arc, south of Japan: an overview. Mar. Georesources Geotech..

[5] Van Dover CL (2019). Inactive sulfide ecosystems in the deep sea: a review. Front. Mar. Sci..

[6] Boschen RE, Rowde AA, Clark MR, Gardner JP (2013). Mining of deep-sea seafloor massive sulfides: a review of the deposits, their benthic communities, impacts from mining, regulatory frameworks and management strategies. Ocean Coast. Manag..

[7] Washburn TW, Turner PJ, Durden JM, Jones DO, Weaver P, Van Dover CL (2019). Ecological risk assessment for deep-sea mining. Ocean Coast. Manag..

[8] Matsui, T., Sugishima, H., Okamoto, N., Igarashi, Y. Evaluation of turbidity and resedimentation through seafloor disturbance experiments for assessment of environmental impacts associated with exploitation of seafloor massive sulfides mining. *Proceedings of the Twenty-eighth. International Ocean and Polar Engineering Conference*. 144–151 (2018).

[9] International Seabed Authority. Recommendations for the guidance of contractors for the assessment of the possible environmental impacts arising from exploration for marine minerals in the Area. https://www.isa.org.jm/documents/isba19ltc8 (2013).

[10] Suzuki K, Yoshida K, Watanabe H, Yamamoto H (2018). Mapping the resilience of chemosynthetic communities in hydrothermal vent fields. Sci. Rep..

[11] Yahagi T, Watanabe H, Ishibashi JI, Kojima S (2015). Genetic population structure of four hydrothermal vent shrimp species (Alvinocarididae) in the Okinawa Trough, Northwest Pacific. Mar. Ecol. Prog. Ser..

[12] Mullineaux LS, Bertness MD, Bruno JF, Silliman BR, Stachowicz JJ (2013). Deep-sea hydrothermal vent communities. Marine community ecology and conservation.

[13] Van Dover CL, German CR, Speer KG, Parson LM, Vrijenhoek RC (2002). Evolution and biogeography of deep-sea vent and seep invertebrates. Science.

[14] Yahagi T, Kayama-Watanabe H, Kojima S, Kano Y (2017). Do larvae from deep-sea hydrothermal vents disperse in surface waters?. Ecology.

[15] Hebert PD, Gregory TR (2005). The promise of DNA barcoding for taxonomy. Syst. Biol..

[16] Iguchi A, Takai S, Ueno M, Maeda T, Minami T, Hayashi I (2007). Comparative analysis on the genetic population structures of the deep-sea whelks *Buccinum tsubai* and *Neptunea constricta* in the Sea of Japan. Mar. Biol..

[17] Goode GB, Bean TH (1879). A catalogue of the fishes of Essex County, Massachusetts, including the fauna of Massachusetts Bay and the contiguous deep waters. Bull. Essex Inst..

[18] Johnson JY (1862). Descriptions of some new genera and species of fishes obtained at Madeira. Proc. Zool. Soc. Lond..

[19] Bate CS (1888). Report on the Crustacea Macrura collected by the Challenger during the years 1873–76. Report on the scientific results of the Voyage of H.M.S. Challenger during the years 1873–76. Zoology.

[20] Folmer O, Black M, Hoeh WR, Lutz R, Vrijenhoek RC (1994). DNA primers for amplification of mitochondrial cytochrome *c* oxidase subunit I from diverse metazoan invertebrates. Mol. Mar. Biol Biotech..

[21] Pilgrim EM, Blum MJ, Reusser DA, Lee H, Darling JA (2013). Geographic range and structure of cryptic genetic diversity among Pacific North American populations of the non-native amphipod *Grandidierella japonica*. Biol. Invasions.

[22] Suyama Y, Matsuki Y (2015). MIG-seq: an effective PCR-based method for genome-wide single-nucleotide polymorphism genotyping using the next-generation sequencing platform. Sci. Rep..

[23] R Core Team. R: A language and environment for statistical computing. R Foundation for Statistical Computing, Vienna, Austria. http://www.R-project.org/ (2020).

[24] Paradis E, Claude J, Strimmer K (2004). APE: analyses of phylogenetics and evolution in R language. Bioinformatics.

[25] Katoh K, Standley DM (2013). MAFFT multiple sequence alignment software version 7: improvements in performance and usability. Mol. Biol. Evol..

[26] Shen W, Le S, Li Y, Hu F (2016). SeqKit: a cross-platform and ultrafast toolkit for FASTA/Q file manipulation. PLoS ONE.

[27] Paradis E (2010). pegas: an R package for population genetics with an integrated–modular approach. Bioinformatics.

[28] Kumar S, Stecher G, Tamura K (2016). MEGA7: molecular evolutionary genetics analysis version 7.0 for bigger datasets. Mol. Biol. Evol..

[29] Darriba D, Posada D, Kozlov AM, Stamatakis A, Morel B, Flouri T (2020). ModelTest-NG: a new and scalable tool for the selection of DNA and protein evolutionary models. Mol. Biol. Evol..

[30] Kozlov AM, Darriba D, Flouri T, Morel B, Stamatakis A (2019). RaxML-NG: a fast, scalable and user-friendly tool for maximum likelihood phylogenetic inference. Bioinformatics.

[31] Ronquist FR, Huelsenbeck JP (2003). MRBAYES 3: Bayesian inference of phylogeny. Bioinformatics.

[32] Puillandre N, Brouillet S, Achaz G (2021). ASAP: assemble species by automatic partitioning. Mol. Ecol. Resour..

[33] Martin, M. Cutadapt removes adapter sequences from high-throughput sequencing reads. EMBnet.journal **17**, http://journal.embnet.org/index.php/embnetjournal/article/view/200/479 (2011).

[34] Rochette NC, Rivera-Colón AG, Catchen JM (2019). Stacks 2: Analytical methods for paired-end sequencing improve RADseq-based population genomics. Mol. Ecol..

[35] Purcell S (2007). PLINK: a tool set for whole-genome association and population-based linkage analyses. Am. J. Hum. Genet..

[36] Jombart T (2008). adegenet: a R package for the multivariate analysis of genetic markers. Bioinformatics.

[37] Goudet J (2013). Hierfstat, a package for R to compute and test hierarchical F-statistics. Mol. Ecol. Notes.

[38] Oksanen, J. *et al.* vegan: Community Ecology Package. R package version 2.5–6. https://CRAN.R-project.org/package=vegan (2019).

[39] Dana JD (1849). Synopsis of the genera of Gammaracea. Am. J. Sci. Arts.

[40] Hansen HJ (1888). Malacostraca marina Groenlandiæ occidentalis Oversigt over det vestlige Grønlands Fauna af malakostrake Havkrebsdyr. Vidensk. Meddel. Natuirist. Foren Kjobenhavn, Aaret.

[41] Van Dover CL (2000). The ecology of deep-sea hydrothermal vents.

[42] Tunnicliffe V (1991). The biology of hydrothermal vents: ecology and evolution. Oceanogr. Mar. Biol. Annu. Rev..

[43] Priede IG, Bagley PM, Smith A, Creasey S, Merrett NR (1994). Scavenging deep demersal fishes of the Porcupine Seabight, north-east Atlantic: observations by baited camera, trap and trawl. J. Mar. Biol. Assoc. U. K..

[44] Causse R, Biscoito M, Briand P (2005). First record of the deep-sea eel *Ilyophis saldanhai* (Synaphobranchidae, Anguilliformes) from the Pacific Ocean. Cybium.

[45] King NJ, Bagley PM, Priede IG (2006). Depth zonation and latitudinal distribution of deep-sea scavenging demersal fishes of the Mid-Atlantic Ridge, 42 to 53°N. Mar. Ecol. Prog. Ser..

[46] Leitner AB, Durden JM, Smith CR, Klingberg ED, Drazen JC (2021). Synaphobranchid eel swarms on abyssal seamounts: largest aggregation of fishes ever observed at abyssal depths. Deep Sea Res. Oceanogr. Res. Part I Pap..

[47] Fishelson L (1994). Comparative internal morphology of deep-sea eels, with particular emphasis on gonads and gut structure. J. Fish. Biol..

[48] Bailey DM (2005). High swimming and metabolic activity in the deep-sea eel *Synaphobranchus kaupii* revealed by integrated in situ and in vitro measurements. Physiol. Biochem. Zool..

[49] Trenkel VM, Lorance P (2011). Estimating *Synaphobranchus kaupii* densities: contribution of fish behaviour to differences between bait experiments and visual strip transects. Deep Sea Res. Oceanogr. Res. Part I Pap..

[50] Raupach MJ, Thatje S, Dambach J, Rehm P, Misof B, Leese F (2010). Genetic homogeneity and circum-Antarctic distribution of two benthic shrimp species of the Southern Ocean, *Chorismus antarcticus* and *Nematocarcinus lanceopes*. Mar. Biol..

[51] Dambach J, Raupach MJ, Leese F, Schwarzer J, Engler JO (2016). Ocean currents determine functional connectivity in an Antarctic deep-sea shrimp. Mar. Ecol..

[52] Dambach J, Raupach MJ, Mayer C, Schwarzer J, Leese F (2013). Isolation and characterization of nine polymorphic microsatellite markers for the deep-sea shrimp *Nematocarcinus lanceopes* (Crustacea: Decapoda: Caridea). BMC Res. Notes.

[53] Ritchie H, Jamieson AJ, Piertney SB (2015). Phylogenetic relationships among hadal amphipods of the Superfamily Lysianassoidea: Implications for taxonomy and biogeography. Deep Sea Res. Part I.

[54] Bowen BW (2014). Phylogeography unplugged: comparative surveys in the genomic era. Bull. Mar. Sci..

[55] Ritchie H, Jamieson AJ, Piertney SB (2017). Population genetic structure of two congeneric deep-sea amphipod species from geographically isolated hadal trenches in the Pacific Ocean. Deep Sea Res. Part I..

[56] Iguchi A (2020). Deep-sea amphipods around cobalt-rich ferromanganese crusts: taxonomic diversity and selection of candidate species for connectivity analysis. PLoS ONE.

[57] Baco AR, Etter RJ, Ribeiro PA, Von der Heyden S, Beerli P, Kinlan BP (2016). A synthesis of genetic connectivity in deep-sea fauna and implications for marine reserve design. Mol. Ecol..

[58] Taylor ML, Roterman CN (2017). Invertebrate population genetics across Earth’s largest habitat: the deep-sea floor. Mol. Ecol..

